# Nanostructural origin of blue fluorescence in the mineral karpatite

**DOI:** 10.1038/s41598-017-10261-w

**Published:** 2017-08-29

**Authors:** Jason Potticary, Torsten T. Jensen, Simon R. Hall

**Affiliations:** 1 Complex Functional Materials Group, School of Chemistry, University of Bristol, Bristol, BS8 1TS United Kingdom; 2 Bristol Centre for Functional Nanomaterials, HH Wills Physics Laboratory, Tyndall Avenue, Bristol, BS8 1TL United Kingdom; 3 Centre for Doctoral Training in Condensed Matter Physics, HH Wills Physics Laboratory, Tyndall Avenue, Bristol, BS8 1TL United Kingdom

## Abstract

The colour of crystals is a function of their atomic structure. In the case of organic crystals, it is the spatial relationships between molecules that determine the colour, so the same molecules in the same arrangement should produce crystals of the same colour, regardless of whether they arise geologically or synthetically. There is a naturally-occurring organic crystal known as karpatite which is prized for its beautiful blue fluorescence under ultra-violet illumination. When grown under laboratory conditions however, the crystals fluoresce with an intense green colour. For 20 years, this difference has been thought to be due to chemical impurities in the laboratory-grown material. Using electron microscopy coupled with fluorescence spectroscopy and X-Ray diffraction, we report here that this disparity is instead due to differences in the structure of the crystals at the nanoscale. The results show that in nature, karpatite has a nanotexture that is not present in the synthetic crystals, which enables different photonic pathways and therefore a blue, rather than green colour whilst undergoing fluorescence.

## Introduction

The optical properties of minerals fundamentally arise from their crystal structure and the spatial arrangement of those crystals across many length scales^1–5^. In the small class of organic hydrocarbon minerals, as is the case in all organic crystals, optical properties are also intimately linked to the relative spatial disposition of molecules within the crystal, with particular polymorphs possessing well-defined optical properties^6–9^. The yellow hydrocarbon mineral karpatite possesses an intriguing property however, in that it fluoresces with a blue colour when illuminated with UV light^10^, whereas an optically, chemically and polymorphically identical synthetic analogue (coronene) is green under the same illumination^11^. Here we show through electron microscopy and X-ray crystallography that it is the texturing of karpatite at the nanoscale that is the cause of this blue fluorescence. It was previously thought that the difference in fluorescence behaviour was due to chemical impurities^12^, but our data show that this is not the case. Our results reveal that the texturing in karpatite results in self-trapped excitons, giving rise to a dominant absorption band at 460 nm that is not present in coronene. These findings highlight the value of applying a suite of advanced methods in the characterisation of rare minerals, particularly where they possess unexplained physical properties. Our report is the first to identify and ascribe dichotomous colour in organic crystals to morphological variances in the solid-state and furthermore suggest that this phenomenon may be general to many other materials. Indeed, this report unambiguously presents the design motifs that must be present in order to account for unexplained colour differences in any organic crystal.

The mineral karpatite (subsequently - K_P_) was first reported in 1955 after being discovered in Zakarpats’ka Oblast in the Ukraine^13^. It is currently known to occur naturally in three locations globally, the Carpathian Mountains, the Tamvatnei mercury deposit in far-east Russia and in San Benito County, CA. in the USA^14^. Historically referred to by a number of names such as ‘Pendeltonite’, ‘Carpathite’ and ‘Karpatite’, it is found as pale-yellow crystalline shards prolifically in cavities between diorite porphyry and argillites (Extended Data Fig. 1). Recent ^13^C isotopic analysis has suggested that the source material for the mineral is organic matter transported via hydrothermal vents from fault-lines^14^. Karpatite is composed of a crystalline arrangement of molecules of coronene (C_24_H_12_) a polyaromatic hydrocarbon (PAH). The molecular structure adopted by coronene has the highest thermal stability for any C_24_H_12_ isomer^13^, whose rigidly planar conformation helps fractionate the solubilised coronene into extremely pure nodes by keeping it separate from surrounding impurities^14^. Commercially available coronene, once purified by sublimation, can be easily crystallised in the laboratory as acicular yellow crystals (subsequently C_N_) which resemble karpatite to the eye.

**Figure 1:**
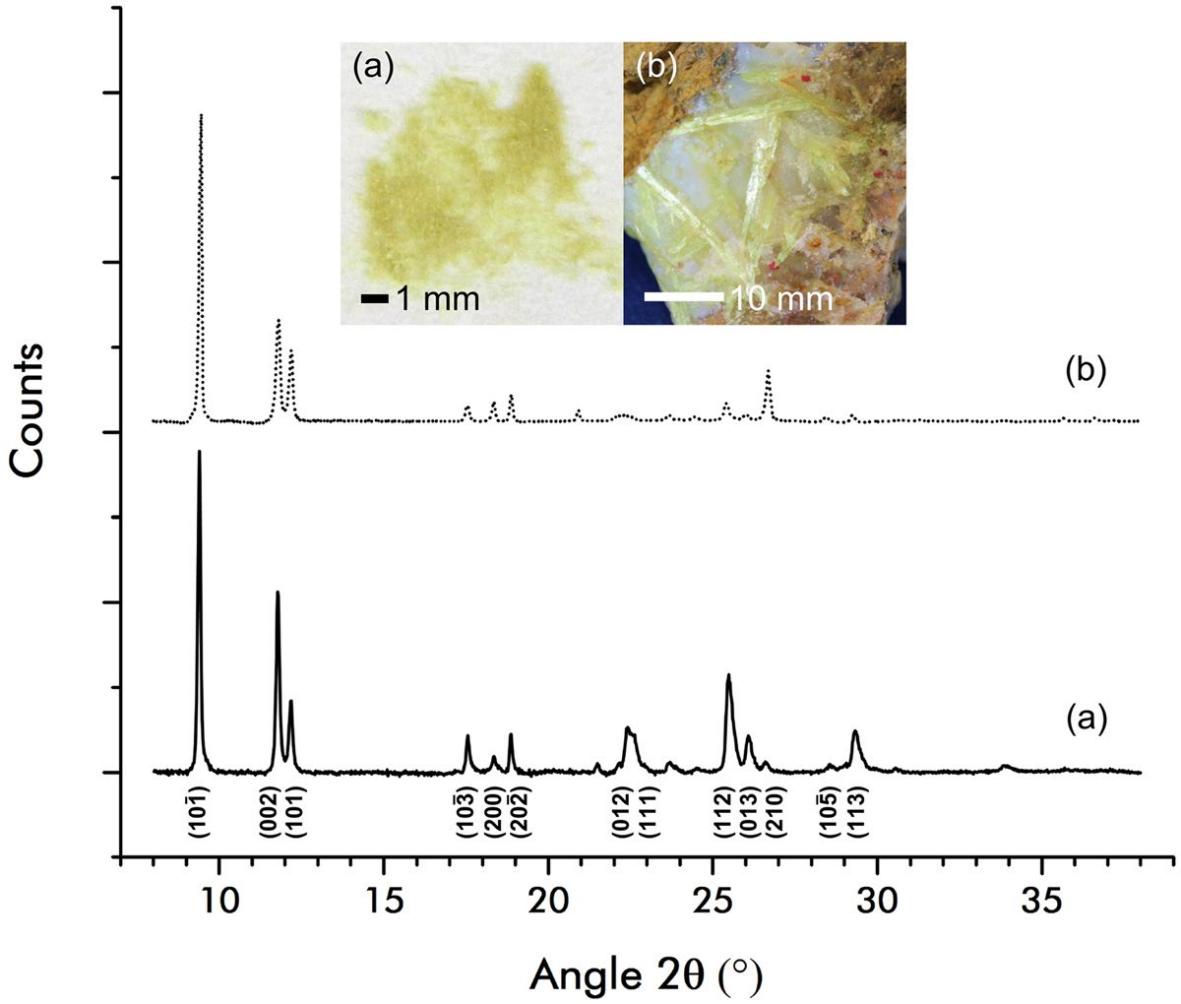
Powder diffraction patterns and images of C_N_ (solid line) and K_P_ (dotted line), major reflections have been labelled. Insets show (**a**) powdered recrystallized C_N_ and (**b**) a vein of K_P_ (yellow crystals) surrounded by quartz (white crystals) and cinnabar (red spots).

The crystal structure of both K_P_ and C_N_ is due to the fact that the coronene molecule has a large delocalised π-system of sp^2^ bonded carbon atoms, with 24 electrons having unimpeded movement around the macro-cycle. This aromaticity lends coronene a rigidly planar conformation within a crystal of which the unit cell is defined as monoclinic, space group P2_1_/n, parameters β = 106.02°, *a* = 10.122 Å, *b* = 4.694 Å, *c* = 15.718 Å and z = 2^15^. Coronene adopts the γ-herringbone structure according to Desiraju and Gavezzoti’s naming convention^16^, based on a 4.70 Å short axis and a nearest neighbour stacking angle of 85.01°. The unit cell of K_P_ was only determined from single crystal data as recent as 2007^14^ and was in agreement with data obtained from lab grown crystals. Figure 1 shows powder XRD patterns of both C_N_ (solid line) and K_P_ (dotted line) along with optical images for comparison. Both patterns can be indexed to the γ-coronene unit cell with some minor differences in reflection intensity.

Analysis of the pXRD pattern of C_N_ confirmed the sample as phase pure P2_1_/n, γ-herringbone crystals (JCPDS card number 12-1611) with little or no amorphous component (Fig. 1 - solid line). Comparison of the K_P_ pattern with the lab-grown C_N_ shows a well-matched pattern with regards to the peak positions (Fig. 1 - dotted line). There is, however, a clear difference in peak intensity, implying a difference in texturing between the two samples. Although crushed, orientational effects are still apparent on the intensity profile of the K_P_ diffraction pattern. Pressing the powder gently onto the XRD sample holder will inevitably cause alignment of the fibres with the substrate.

Historically, the colour of crystallised coronene, whether C_N_ or K_P_, has been described in a variety of ways. Whereas almost all lab-prepared coronene is referred to as having a green appearance under UV illumination, Karpatite has been reported as being ‘electric blue’ or even ‘blue-green’^17^ under the same conditions. Although known to be identical chemically and crystallographically, these terms have been used interchangeably throughout the literature, curiously with no distinction or attempt to account for the clear difference in colour between K_P_ and C_N_. Previous investigations on the optical response of PAHs suggest that the cycle stack overlap is the major contributing factor to the strong fluorescence under UV illumination^18^; indeed, recent work would appear to support this claim^19^. However, as polymorph dependant variations in fluorescence are, by definition, restricted to changes in the fundamental unit cell of the PAH, differences between two materials that share the same crystal structure such as K_P_ and C_N_ crystals cannot be explained this way. As photon emission and reabsorption is a well-known phenomenon in PAH crystals^20^, here we identify and quantify the difference in the optical behavior of C_N_ and K_P_ as being the result of macro-structural differences in the two crystalline forms, enabling quite distinct emission and reabsorption pathways.

**Figure 2:**
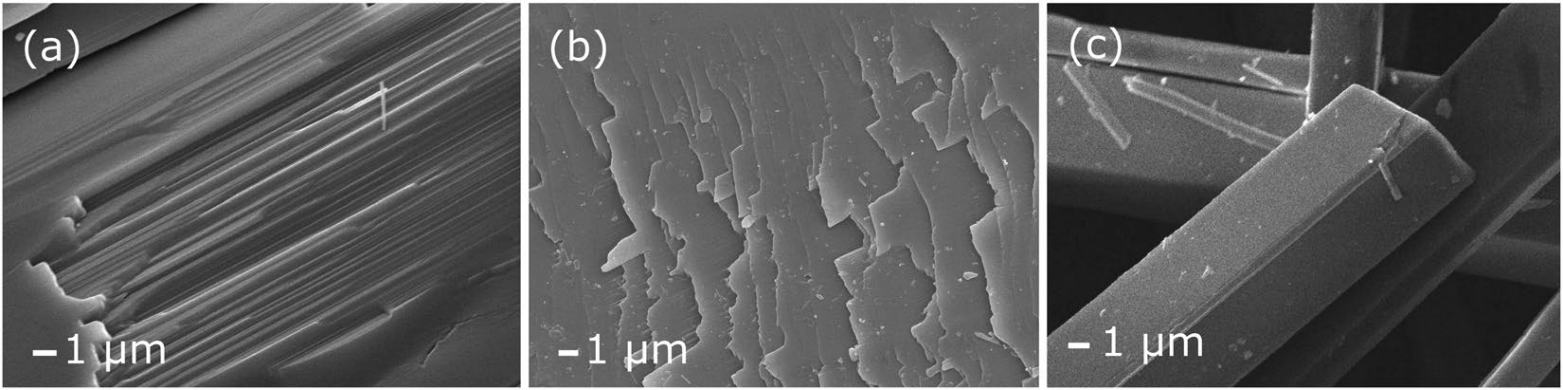
SEM micrographs of native K_P_ and C_N_ purified by sublimation. (**a**) Detail of the broken karpatite surface and (**b**) the layering of karpatite exposed at the end of the layered structure. (**c**) An image of a lab grown C_N_ viewed at the same magnification.

Optical microscopy of C_N_ reveals high aspect-ratio needles with parallel sides and truncated ends, consistent with the monoclinic γ-polymorph known to show favourable growth along the short, *b*-axis. When viewed under a polarised-light microscope, parallel extinction was observed indicating that each needle was indeed a single crystal. In order to determine crystallite structure and shape, scanning electron microscopy was used. Figure 2(a) and (b) show starkly that K_P_ possesses a fibrous/layered structure, with layers having a thickness of ~100 nm. These layers can be seen clearly throughout the entire sample. Figure 2(b) shows an image of the end of a fibre as the layers cause it to taper off. In contrast, Fig. 2(c) shows an SEM micrograph of C_N_ at the same magnification with no layering visible and no fraying of needle ends. These high-aspect ratio needles have polygonal-defined cross-sections and crystal faces appear smooth and edges are angular.

**Figure 3:**
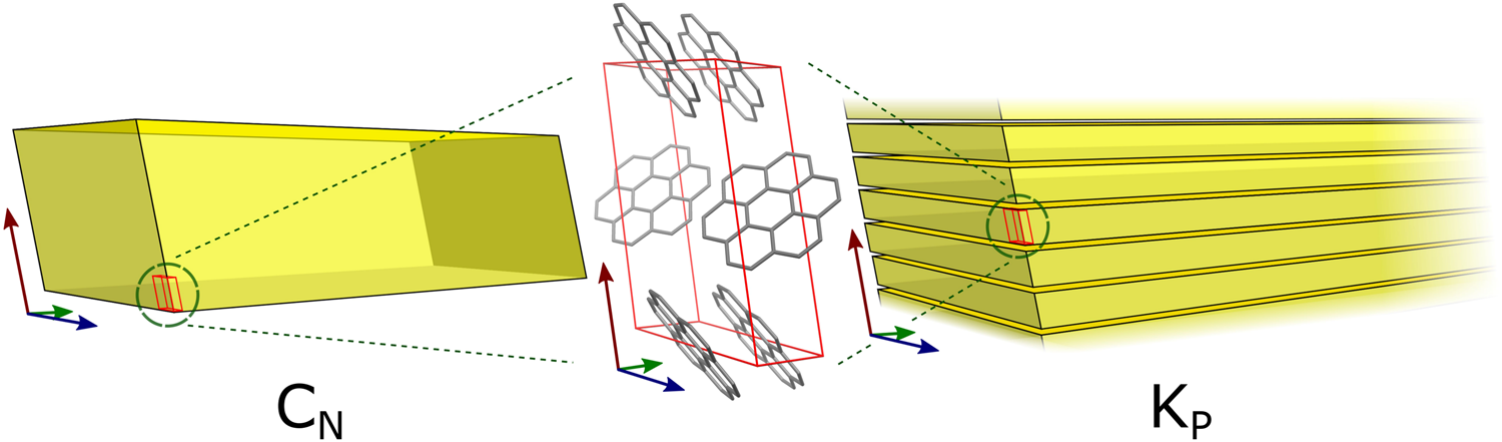
Orientation of the p21/n unit cell of coronene in both C_N_ (left) and K_P_ (right). Blue, green and red arrows represent the *a-*, *b-* and *c-*axis directions respectively.

Single crystal X-ray diffraction of C_N_ confirms the unit cell was monoclinic with space group P2_1_/n as expected. Unit cell parameters were β = 106.29 °, *a* = 10.03 Å, *b* = 4.68 Å, *c* = 15.58 Å and z = 2. Stacking distance was calculated to be 3.38 Å. In addition, it is determined that the major growth direction of the C_N_ needles is also along the short, *b*-axis (Fig. 3).

**Figure 4:**
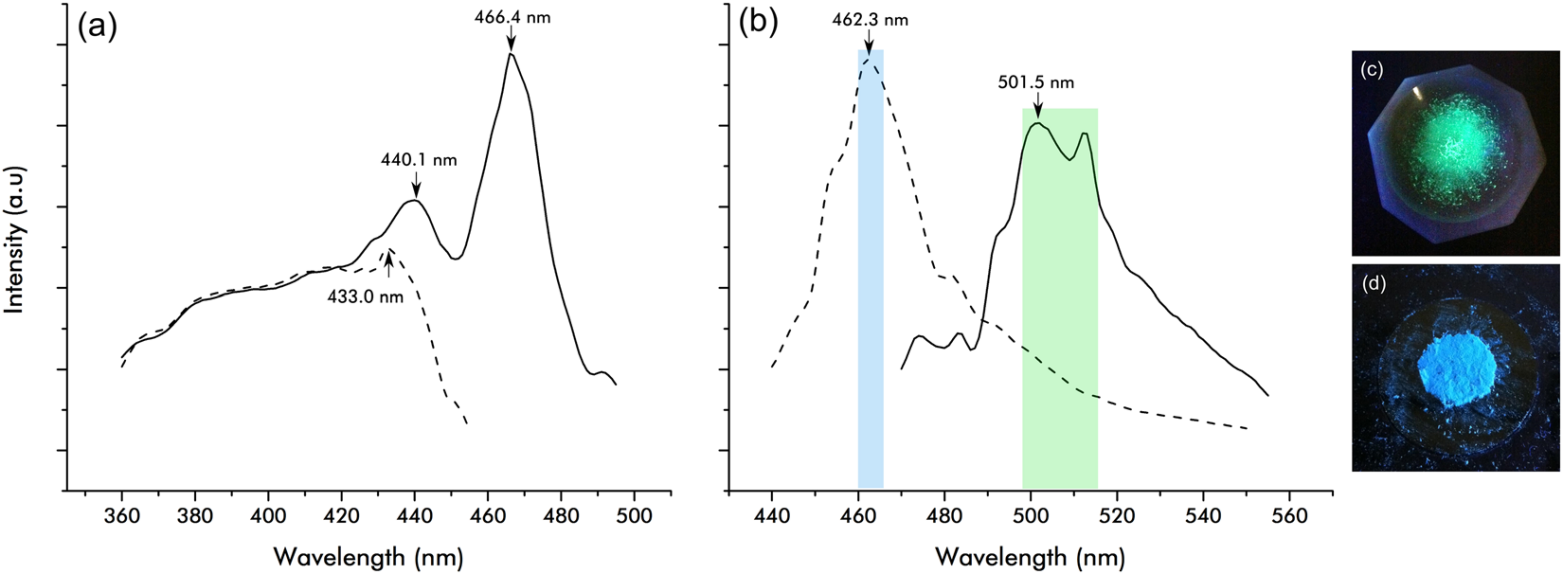
Solid state fluorescence of C_N_ (solid line) and K_P_ (dotted line). (**a**) Excitation and (**b**) emission wavelengths were taken using λ_max_ from the corresponding data (C_N_ ex = 466.4 nm/em = 501.5 nm, K_P_ ex = 433.0 nm/em = 462.3 nm). The inset images show (**c**) C_N_ and (**d**) K_P_ photographed under shortwave UV illumination.

In order to determine the molecular absorbance of both forms, UV-vis absorption measurements were taken of both types of crystal after dissolution in toluene (Extended Data Fig. 2). Both C_N_ and K_P_ produced results that were indistinguishable from each other after a background of pure solvent was subtracted. Absorbance peaks at 341 nm, 326 nm, 305 nm and 293 nm agree with the literature values for coronene^21, 22^. Having concordant data between coronene and karpatite in solution confirms that molecularly, the two materials are identical. It is in the solid state, therefore, that we must look to uncover the cause of the materials’ differing properties. As stated above, after being illuminated with UV light, K_P_ emits a brilliant blue colour (Fig. 4(d)), whilst C_N_ appears green in colour under the same conditions (Fig. 4(c)).

Although coronene has been shown to emit at different wavelengths previously, this is only in solution and bound to other materials^22^. As C_N_ and K_P_ are chemically and crystallographically identical, this implies that the difference in the solid state UV response must result from the observed differences in the macroscopic structure (Fig. 2). To measure the difference in UV response quantitatively, both C_N_ and K_P_ were analysed via UV-vis spectrophotometry in reflectance mode. For diffuse reflectance measurements, the samples were scanned over the range 800 to 240 nm. Extended Data Fig. 3 shows both the raw diffuse reflectance as a function of λ and the analogous absorbance calculated using the Kubelka-Munk^23^ function plotted against energy. The polycrystalline nature of the materials does not allow for a direct comparison of the relative intensities via diffuse reflectance measurement, however it can clearly be seen that the responses of both solids are plainly dissimilar, as evidenced from the starkly different lineshapes. In the case of K_P_ (dashed line), reflectance begins to drop sharply at 471 nm to 22% at 432 nm. In C_N_ however, there is a much more gradual decrease in reflectance from 792 nm to 467 nm. Both samples display a similar reflectance from 432 nm to 230 nm with slight differences between 432 nm and 361 nm. The optical band gap for C_N_ here is calculated as 2.403 eV, which matches closely the literature value of 2.41 eV.^24^ In contrast, K_P_ shows an optical band gap of 2.743 eV, a difference of 0.340 eV from the lab-grown crystal of C_N_. Figure 4(a) and (b) show the excitation and emission profiles of both C_N_ and K_P_. The emission profiles for each crystal are markedly different. K_P_ has a maximum emission at 462.3 nm which is in the blue region of visible light, whereas C_N_, has emissions at 501.5 nm and 512.2 nm which is deep within the green. The excitation profile of K_P_, for an emission of 462.3 nm, increases fairly evenly with wavelength until 433.0 nm is reached, at which point it sharply drops away. Across the same range, C_N_ with an emission at 501.5 nm resembles the lineshape for K_P_ until, unlike K_P_, the intensity diminishes. The excitation profile keeps rising until 440.1 nm where it drops again before rising to its maximum intensity at 466.4 nm. It is important to note, upon analysis of these results, intense peaks in the excitation of C_N_ at 466.4 nm and the emission by K_P_ at 462.3 nm are of similar shape at a similar wavelength. An energy of 2.66 eV −2.68 eV (466 nm −462 nm) is being emitted from the layered crystal of K_P_ but not the non-layered C_N_. If this layering in K_P_ is the fundamental cause of the differences in the emission profiles, it would therefore naturally be expected that K_P_ crystals without this nano-texture would emit in the green wavelength region. To determine if this was indeed the case and to rule out the role of any potential polyaromatic impurities, some K_P_ was dissolved in toluene, after confirmation of its expected blue emission, and recrystallized by slow evaporation. The resulting crystals were prismatic and indiscernible from C_N_ via SEM and fluorescence spectroscopy (Extended Data Fig. 4). Furthermore, sublimation of K_P_ crystals resulted in the deposition of crystals in the cooler region of the tube that exhibited a fluorescence spectroscopy profile similar to K_P_ crystals, but with the addition of peaks in the C_N_-like green region of the emission spectrum at 463, 482 and 527 nm (Extended Data Fig. 5). This would suggest that the sublimation resulted in a mixture of layered and non-layered morphologies, which indeed was the case, as evidenced by SEM images of the sublimed crystals. (Extended Data Fig. 6). These data confirm that the discrete nano-texturing of K_P_ is the most likely cause of the blue fluorescence. In order to confirm this, we need to consider what mechanism is giving rise to the blue fluorescence in K_P_, with the goal of determining how charge transport differs between K_P_ and C_N_. As non-charged molecules, excitonic charge transfer between molecules in PAH crystals, is known to occur along the π-stack, which in the case of C_N_ is typically the *b*-direction, along the length of the needle^25, 26^. It has previously been calculated that this charge transfer between coronene molecules requires 2.68 eV^24^ which is extremely close to the 2.66 eV measured experimentally in K_P_ via UV-vis (the location of the similar band in Fig. 4 mentioned earlier, 2.66–2.68 eV). If charge transfer is indeed causing this optical difference between K_P_ and C_N_, then it is clear that the K_P_ *c*-axis (corresponding to the thickness of the layers in K_P_), must also be the primary route for this 2.66 eV charge transfer, as this is not a route available in C_N_ due to the lack of nano-texturing in C_N_. It follows that electron hopping in K_P_ involves a significant fraction of movement across the stacks, via edge-face interactions. Another way of confirming that charge transfer excitons are responsible for the colour change is to look at the spectral response of thin (approximately 1 μm thickness) plate-like C_N_ crystals. Previous studies have shown that these thin C_N_ crystals do indeed possess a similar spectrum to that seen in K_P_ sample, with the dominant absorption band at approximately 460 nm in K_P_ and thin C_N_ attributed to the presence of self-trapped excitons^12, 27^. Calculations performed by Shinozuka and Toyozawa of the relaxed state energy of one, two and three-dimensional organic crystals indicate that, for an infinite three-dimensional crystal lattice, the free exciton state is stable, with a large potential barrier preventing the transition to the trapped state. However, with a finite number of lattice sites and reduced dimensionality, the free state possesses higher energy and thus a free exciton is more likely to transition to the lower-energy self-trapped state^28^. Hence, it appears that the lack of this band in C_N_ crystals is due to the greater potential barrier separating the free and self-trapped exciton states, compared to that in nanotextured K_P_ crystals. In contrast to other organic crystals, coronene possesses a deeper self-trapped state^29^, which is why it an ideal model for studying the effects of morphological change on the alteration of excitonic luminescence.

In conclusion, we have determined that despite being compositionally identical, lab-grown crystalline coronene and naturally-occurring karpatite exhibit different fluorescence profiles due to nano-texturing of karpatite. Furthermore, we have shown that it is the alteration of the charge transfer mechanism that is concomitant with this texturing that explains the change in fluorescence. Our findings show that through the application of a suite of advanced analytical techniques, a new and deeper understanding of the physical behaviour of rare minerals with unexplained properties can be achieved. As this study represents the first proof that colour change in organic crystals can be a solid-state morphological phenomenon, we believe that our interrogative method can be applied to many other organic crystal systems to potentially uncover exotic charge transfer pathways in semiconductors, field-effect transistors and organic superconductors.

## Methods

### Crystal Preparation

Coronene (C_N_) was synthesised using (97%) purchased from Sigma-Aldrich UK and twice purified by sublimation under vacuum after recrystallization from toluene. The resulting crystals were yellow needles of various lengths up to a maximum of 5 mm. The naturally formed karpatite was reclaimed by gentle excavation of the soft crystalline region, embedded in surrounding quartz, using a PTFE coated spatula to avoid metallic contamination. The pale-yellow flakes were separated from any debris under an optical microscope, no further purification was performed.

### Crystallography

Powder x-ray diffraction (pXRD) data were gathered using a Bruker D8 Advance diffractometer (Cu-Kα radiation - wavelength of 1.5418 Å) with a PSD LynxEye Detector. Step size was 0.0411 2θ and hold time was 1.5 s. Both C_N_ and K_P_ were only subject to a gentle crushing prior to analysis. Both samples were mounted on a low-background sample holder with silicon wafer.

### UV-vis spectroscopy

UV-vis spectroscopy was taken using a Perkin-Elmer Lambda 25 UV/vis spectrophotometer. Samples in solution were dissolved at a known concentration, and dispensed into a 1 cm path-length, quartz cuvette and tested for absorbance between 280 and 360 nm. UV reflectance was obtained in the solid state by mounting the crystalline sample on a barium sulphate substrate behind a quartz window. Using BaSO_4_ as a reference, the diffuse reflectance of the sample was recorded. This reflectance was converted to analogous solid absorption using the Kubelka-Munk function^11^.

### Fluorescence spectroscopy

Measurements were obtained after mounting crystalline samples on the end of a quartz plate using paraffin oil. The quartz plate, cut at an 8° angle allowed for emitted light to be detected but not directly reflect the incident beam. Initial excitation wavelengths were informed by solid state UV-vis results after which emission peaks were identified.

### SEM

All Scanning Electron Microscopy (SEM) samples were prepared by mounting on 7 mm aluminium stubs using a sticky carbon pad. These were then sputter coated with a 15 nm of silver. Images were acquired using a JEOL JSM 6330 F high-resolution SEM with a field emission gun and captured using a range of working distances and accelerating voltages.

### Data Availability

Data are available at the University of Bristol data repository, data.bris, at https://doi.org/10.5523/bris.3v4w0ka3q7lqg27gznygvh0s02. 

## Electronic supplementary material

Supplementary information
