# Supplementary material for: Nanostructural origin of blue fluorescence in the mineral karpatite
Source: Sci Rep. 2017 Aug 29;7:9867. doi: 10.1038/s41598-017-10261-w (PMC5575318; doi:10.1038/s41598-017-10261-w)
Supplement: Supplementary file 1 — Supplementary information [file 41598_2017_10261_MOESM1_ESM.pdf]

Nanostructural origin of blue fluorescence in the mineral karpatite

Authors: Jason Potticary[1,2], Torsten T. Jensen[1,3] and Simon R. Hall[1*]

Extended Data

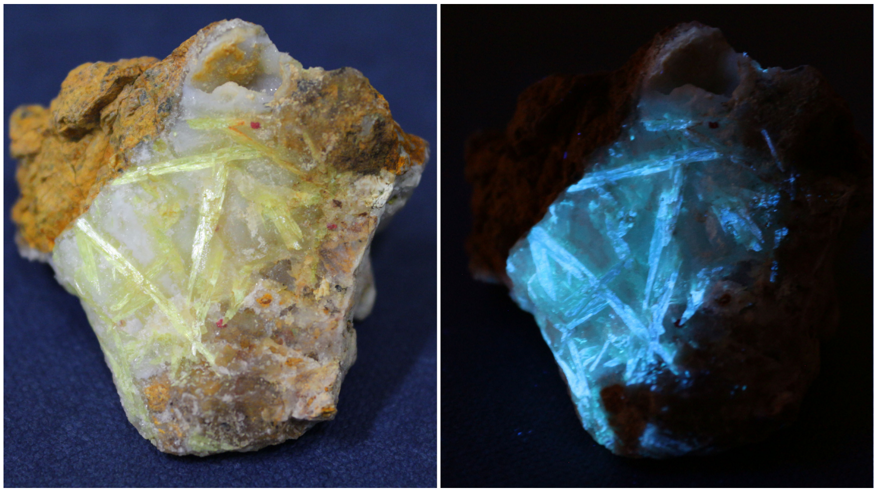

Extended Data Fig. 1. Karpatite as found embedded in quartz in day light (left) and under UV illumination (right).

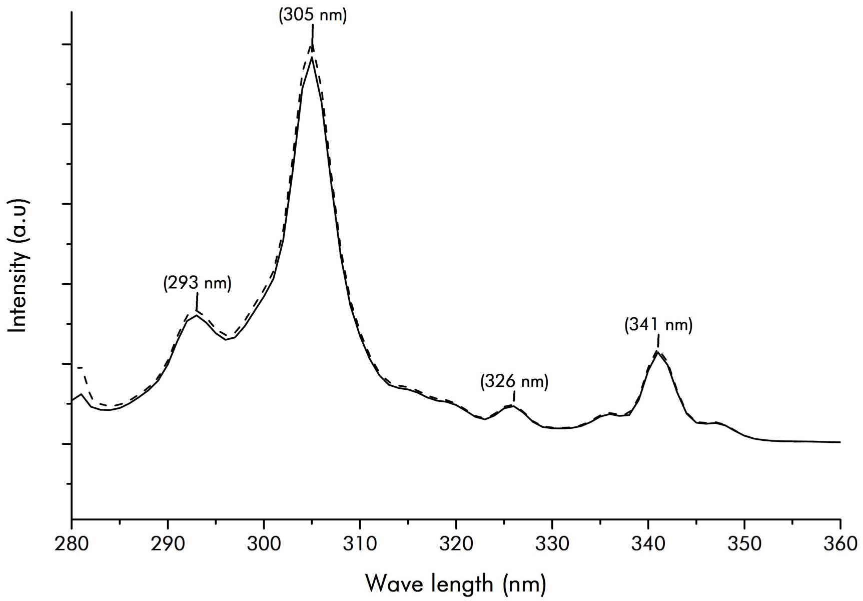

Extended Data Fig. 2. UV-vis spectrum of both C_N (solid line) and K_P (dashed line) dissolved in toluene.

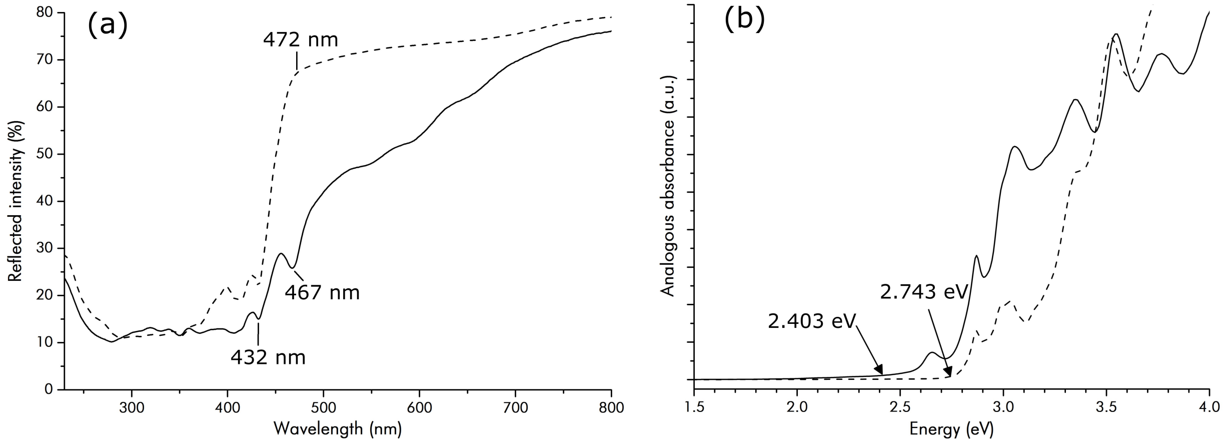

Extended Data Fig. 3. Solid state UV-vis spectroscopy of coronene. (a) Diffuse reflectance absorbance of C_N (solid line) and K_P (dashed line) and (b) Diffuse reflectance data as processed using the Kubelka-Munk function, showing lowest energy absorbance.

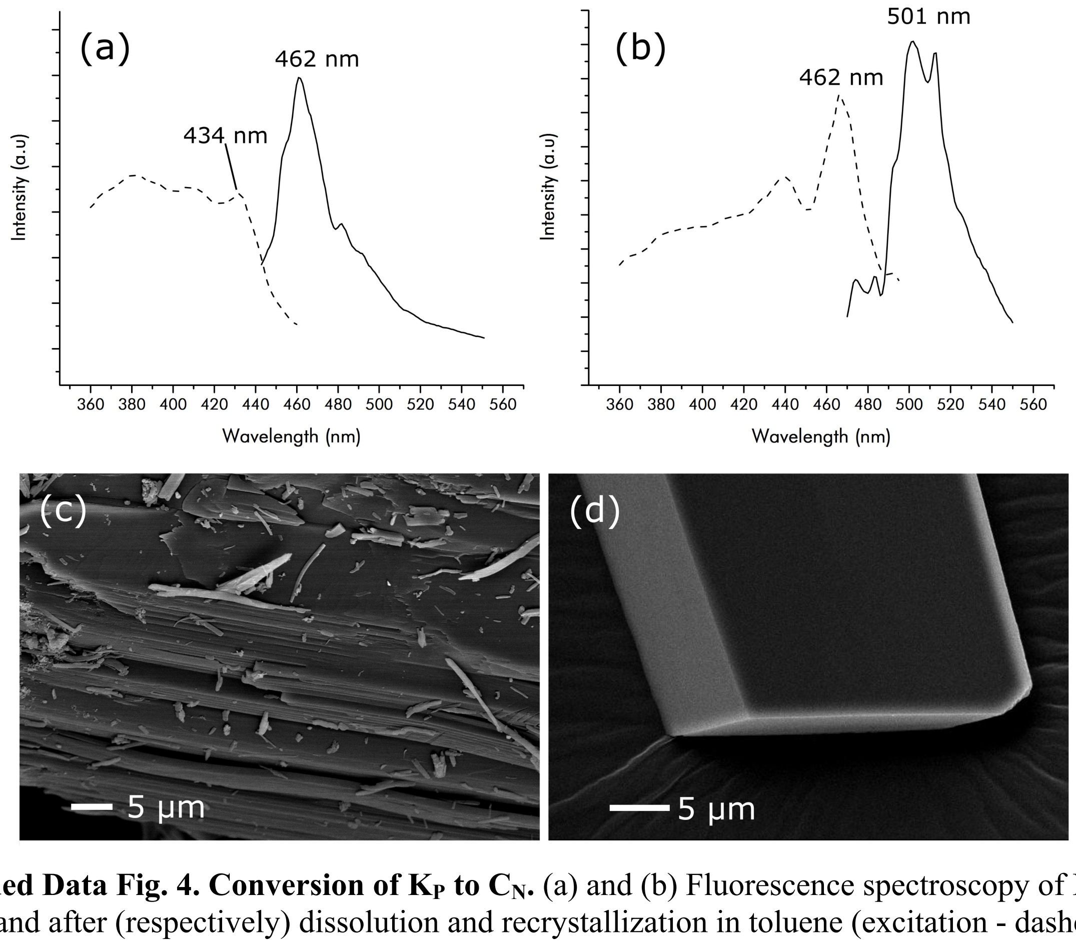

Extended Data Fig. 4. Conversion of K_P to C_N. (a) and (b) Fluorescence spectroscopy of K_P before and after (respectively) dissolution and recrystallization in toluene (excitation - dashed line and emission - solid line). (c) and (d) SEM micrographs of crystals, below their corresponding spectra.

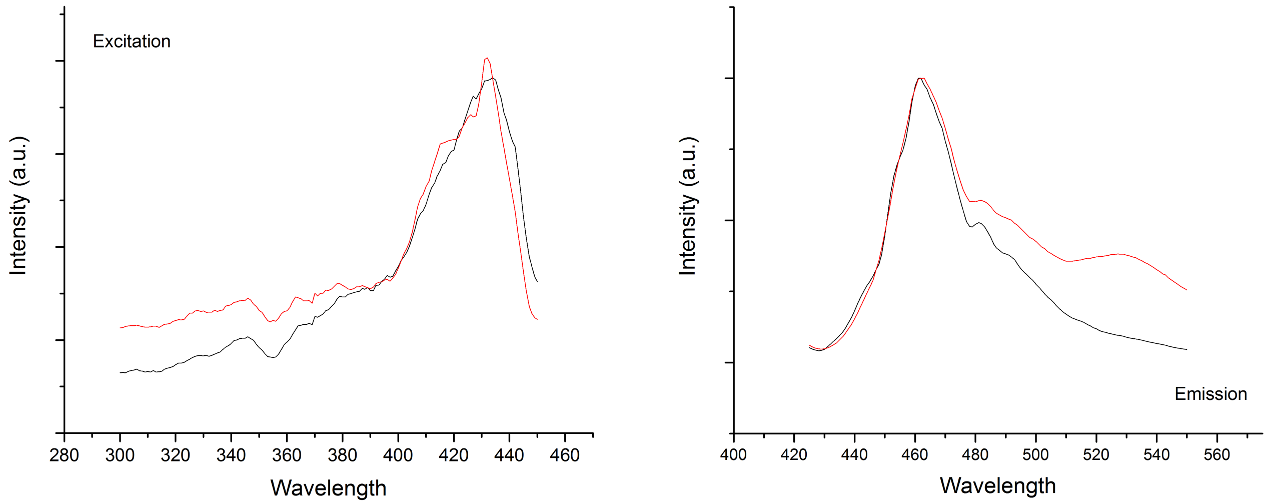

Extended Data Fig. 5. Excitation measurements of karpatite (black line) and resublimed karpatite (red line) were taken by measuring emission at 463 nm and exciting between 300 and 450 nm. 5 measurements were taken of each and an average obtained. Emission measurements of resublimed karpatite were taken by exciting at 410 nm and measuring emission between 425 nm and 550 nm. 5 measurements were taken of each and an average obtained.

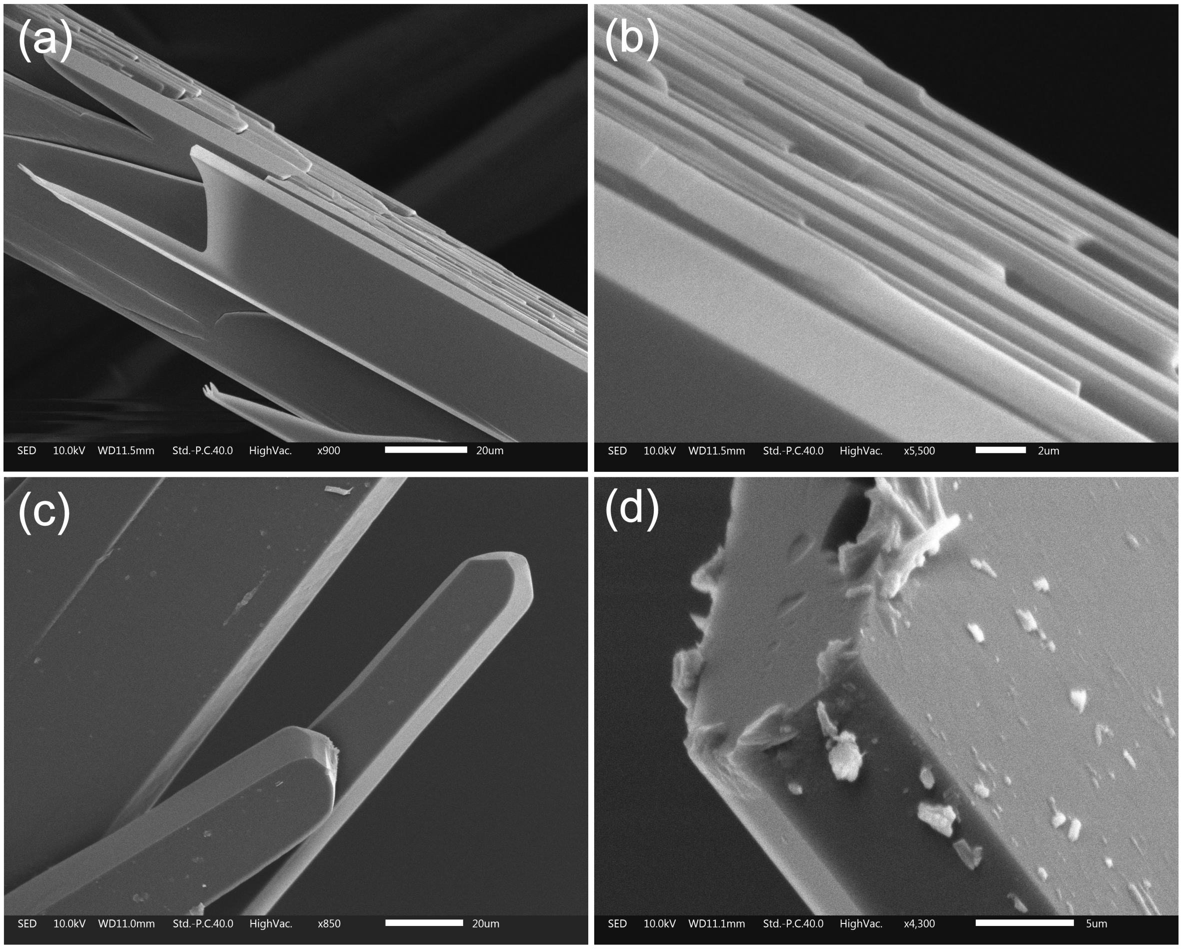

Extended Data Fig. 6. SEM images of sublimed karpatite crystals. Images (a) and (b) show texturing of the crystals, whereas (c) and (d) show no texturing. All crystals in this figure are from the same experiment. Scale bar in (a) and (c) is 20 μm, in (b) 2 μm and in (d) 5 μm.
